# Impact of diabetes mellitus on indeterminate results of the QuantiFERON TB Gold In-Tube test: A propensity score matching analysis

**DOI:** 10.1371/journal.pone.0181887

**Published:** 2017-07-21

**Authors:** Hong-Joon Shin, Tae-Ok Kim, Hyung-Joo Oh, Ha-Young Park, Jin-Sun Chang, Seong Ahn, Yu-Il Kim, Sung-Chul Lim, Yong-Soo Kwon

**Affiliations:** Department of Internal Medicine, Chonnam National University Medical School, Gwangju, Republic of Korea; Chinese Academy of Medical Sciences and Peking Union Medical College, CHINA

## Abstract

**Background:**

The sensitivity of interferon-gamma release assays (IGRAs) in the detection of *Mycobacterium tuberculosis* infection could be affected by conditions of immune dysregulation. For this reason, diabetes mellitus (DM) may increase the frequency of indeterminate results of IGRAs. However, there have been inconsistent reports of role of DM on indeterminate IGRA results.

**Methods:**

We retrospectively reviewed all patients who underwent QuantiFERON-TB Gold In-Tube testing (QFT-GIT) at Chonnam National University Hospital. We collected the clinical and laboratory data of these patients.

**Results:**

Of all 3,391 subjects, 1,265 (37.3%) had a positive QFT-GIT result, 266 (7.8%) had an indeterminate result, and 1,860 (54.9%) had a negative result. The mean age was 54.8 ± 18.1 years and 55.0% of the patients were male. There were 512 (15.1%) patients with DM. Multivariable analysis revealed that systemic corticosteroid use, tuberculosis, lymphocytopenia, low serum albumin, and high serum C-reactive protein (CRP) levels were significantly associated with indeterminate QFT-GIT results. However, DM was not associated with indeterminate QFT-GIT results (adjusted odds ratio, 0.98; 95% confidence interval, 0.69–1.41; P = 0.939). After propensity score matching, DM was not associated with indeterminate results of QFT-GIT.

**Conclusion:**

In this large cohort study, DM does not affect the incidence of indeterminate results of QFT-GIT.

## Introduction

Interferon-γ (IFN-γ) release assays (IGRAs) are immunologic tests that can help clinicians diagnose tuberculosis (TB) and latent *Mycobacterium tuberculosis* (*Mtb*) infection by demonstrating the immunologic response to *Mtb* antigens [1]. The QuantiFERON-TB Gold In-Tube test (QFT-GIT) is an enzyme-linked immunosorbent assay (ELISA) that quantifies the IFN-γ response of fresh whole blood to a cocktail of *Mtb* antigens (early secretory antigenic target-6 [ESAT-6], culture filtrate protein-10 [CFP-10], and TB7.7), and it has been used widely. However, indeterminate results could limit the usefulness of this test and cause confusion for clinicians in the diagnosis of *Mtb* infection. Moreover, the rate of indeterminate results is relatively high; a recent meta-analysis showed that the incidence of indeterminate QuantiFERON-TB Gold test results ranged from 3% to 21%. This included a pooled rate of 2.1%, which increased to 4.4% in immunosuppressed patients [2].

Diabetes mellitus (DM) is an important co-morbidity in patients with tuberculosis (TB) because it can increase the risk of active TB [3, 4], have severe clinical manifestations, and cause worse outcomes [5, 6]. DM can also cause dysregulation of the immune system, resulting in altered levels of cytokines and chemokines [7, 8]. However, there have been inconsistent reports of IFN-γ levels in response to *Mtb* in patients with TB and latent TB infection. Studies in patients with TB showed that those with DM had a higher response to a purified protein derivative from *Mtb* [9] and *Mtb* antigens [10], but no difference in the response to an *Mtb* sonicate [11] compared with patients without DM. However, another study in patients with latent TB infection showed that the response to *Mtb* antigens was decreased in patients with DM compared with that in patents without DM [12]. Furthermore, a recent study indicated that TB patients with DM were over 4 times more likely to have indeterminate QFT-GIT results using univariate analysis, although the effect was lost after adjusting for confounders [13]. In this study, we evaluated whether an indeterminate result of QFT-GIT is affected by DM.

## Methods

### Study population

We retrospectively reviewed all patients aged over 18 years who underwent QFT-GIT testing at Chonnam National University Hospital between January 2013 and December 2014.

### Immunocompromised medical conditions

Immunocompromised patients were defined as those who had comorbidities, such as: DM, chemotherapy for an underlying malignancy at the time of QFT-GIT, solid organ transplant or bone marrow transplant, renal replacement therapy for chronic renal failure, human immunodeficiency virus (HIV) infection, and advanced liver cirrhosis of Child-Pugh class C. Patients receiving daily administration of immunosuppressive agents (at least 15 mg of prednisone per day for more than 1 month, or combination therapy with low dose corticosteroids and other immunosuppressive agents, including azathioprine, mycophenolate, methotrexate, cyclosporine, or cyclophosphamide) were also defined as immunocompromised. DM was defined by a history of DM with prior use of antidiabetic medications.

### Diagnosis of TB

TB was diagnosed on the basis of *Mycobacterium tuberculosis* identification by culture or polymerase chain reaction from clinical specimens or clinical, radiological, or histological findings compatible with TB and responses to TB treatment.

### Assessment of clinical and laboratory findings

Laboratory data included lymphocyte and platelet counts, hemoglobin, albumin, C-reactive protein (CRP), and serum glucose levels. HIV was detected using screening with an Architect HIV Ag/Ab Combo Kit (Abbott Laboratories, Abbot Park, IL, USA) to determine the presence of the HIV antibody and/or antigen. The results were confirmed using an additional HIV Western blot (HIV BLOT 2.2 Western Blot Assay, MP Diagnostics, Asia Pacific Pte Ltd., Singapore). Anemia was defined according to the World Health Organization (WHO) guidelines as a baseline hemoglobin level <13 g/dL in men or <12 g/dL in women [14]. Lymphocytopenia was defined as a blood lymphocyte count <1.0 × 10^9^/L.

### QuantiFERON-TB Gold In-Tube test

The QFT-GIT test was performed according to the manufacturer’s instructions. Briefly, the test consisted of 3 blood collection tubes: a nil control tube (negative control: whole blood without antigens or mitogen), a mitogen control tube (positive control: whole blood with phytohemagglutinin), and a TB antigen tube (whole blood with a synthetic peptide cocktail simulating the *Mtb-*specific antigens, including ESAT-6, CFP-10, and TB7.7). The blood tubes were incubated for 20 hours at 37^°^C. The concentrations of IFN-γ were measured using an ELISA, which was either performed manually or using an automated microplate processor (Evolis Twin Plus system; Bio-Rad Laboratories, Hercules, CA, USA)

### Definitions of indeterminate result

The QFT-GIT results for each patient were interpreted according to the manufacturer’s criteria. Briefly, the QFT-GIT result was defined as positive if the IFN-γ level of Nil was ≤ 8.0 IU/mL and that of TB antigen minus Nil was ≥ 0.35 IU/mL and ≥ 25% of Nil value. A negative result was defined if the IFN-γ level of Nil was ≤ 8.0 IU/mL, that of Mitogen minus Nil was ≥ 0.5 IU/mL, and that of TB antigen minus Nil was < 0.35 IU/mL or < 25% of Nil value. The results were reported as indeterminate if the IFN-γ level of Nil was ≤ 8.0 IU/mL, that of TB antigen minus Nil was< 0.35 IU/mL or ≥ 0.35 IU/mL and < 25% of Nil value, and Mitogen minus Nil was < 0.5 IU/mL (positive control failure) or if the IFN-γ level of Nil was > 8.0 IU/mL (negative control failure). Positive and negative results for QFT-GIT were combined into 1 variable termed “determinate”.

### Statistical analysis

All data are expressed as mean or standard deviation or as numbers and percentages. The Pearson’s χ^2^ or Fisher’s exact tests were used for categorical variables to compare between determinate and indeterminate groups. The Student’s t-test were used for continuous variables to compare between determinate and indeterminate groups. To identify factors associated with indeterminate QFT-GIT results, logistic regression analysis was used in univariate analysis. Subsequently, multivariable logistic regression analysis was carried out, with inclusion of variables with P values of <0.25 in univariate analysis with enter method. We assessed the interactions between variables which were introduced the multivariable logistic regression model. There was significant interaction between DM and malignancy undergoing chemotherapy (P = 0.038). Therefore, we decided not to introduce the variable with malignancy undergoing chemotherapy to the final multivariable logistic regression model. We also measured the variance inflation factor (VIF) to identify the existence of collinearity in the variables of multivariable logistic regression analysis. We considered as a presence of collinearity if VIFs of variables were > 5.

To evaluate influence of DM on indeterminate QFT-GIT result compared to determinate QFT-GIT result, we matched patients according to covariates of age, sex, rheumatic disease, malignancy undergoing chemotherapy, solid organ transplant, bone marrow transplant, chronic renal failure with renal replacement therapy, advanced liver cirrhosis, HIV infection, systemic corticosteroid or immunosuppressive agent use, diagnosis of TB, lymphocytopenia, anemia, and serum albumin and CRP levels. Propensity score matching was performed with one-to-one nearest neighbor matching without replacement. The caliper was 1.0 and the absolute standardized differences of the mean were within 0.1 for all variables. All subsequent analyses were performed in the matched sample, using methods appropriate for the analysis of matched data. The Paired t-test was used for continuous variables and the McNemar’s test was used for categorical variables to compare between DM and non-DM groups after matching [15].

All statistical analysis was performed using SPSS 21.0 (SPSS Inc., Chicago, IL). Propensity score matching was performed using IBM SPSS 21.0 and R version R2.14.2 (R Foundation for Statistical Computing, Vienna, Austria). P < 0.05 was considered statistically significant.

### Ethics statement

The protocol of this study was approved by the Chonnam National University Hospital Institutional Review Board. Informed consent was waived because of retrospective nature of this study, and patient information was anonymized and de-identified prior to analysis. (IRB number, CNUH-2017-169).

## Results

### Patient characteristics

**Fig 1:**
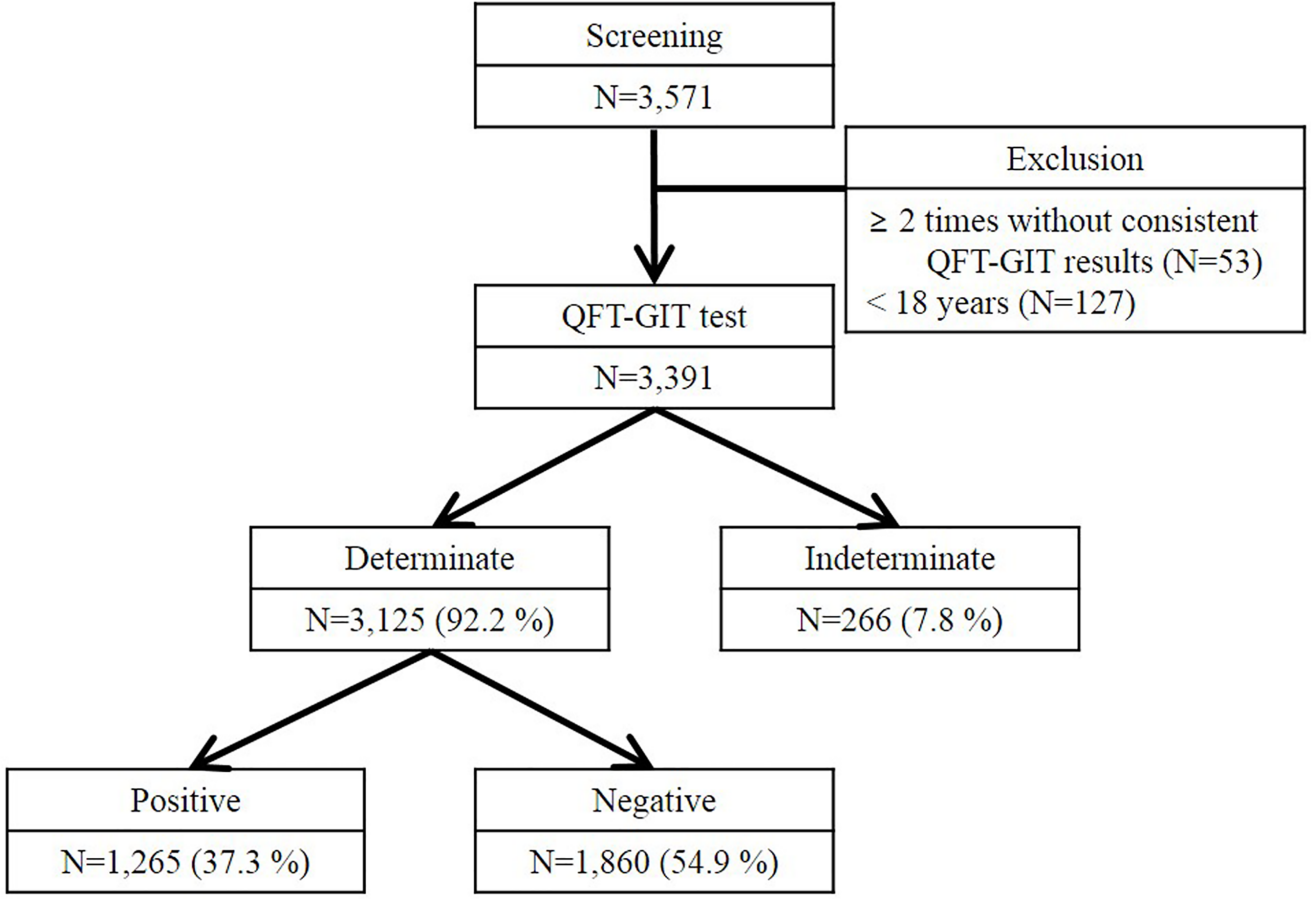
Flow chart of the study protocol. We screened 3,571 subjects who underwent QuantiFERON-TB Gold In-Tube testing (QFT-GIT) testing more than once. Of these subjects, patients who were under 18 years old (n = 127) or who underwent QFT-GIT more than twice without consistent results (n = 53) were excluded. After exclusion, 3,391 patients were enrolled in the study in which 1,265 (37.3%) had a positive QFT-GIT result, 266 (7.8%) had an indeterminate result, and 1,860 (54.9%) had a negative result.

Fig 1 shows the flowchart of this study. From January 2013 to December 2014, 3,571 patients underwent QFT-GIT testing more than once. We excluded patients who were under 18 years old (n = 127) or who underwent QFT-GIT more than twice without consistent results (n = 53). Of all 3,391 patients, 1,265 (37.3%) had a positive QFT-GIT result, 266 (7.8%) had an indeterminate result, and 1,860 (54.9%) had a negative result.

The baseline characteristics of each group are presented in Table 1. The mean patient age was 54.8 ± 18.1 years and 1,864 patients (55.0%) were men. A total of 512 patients (15.1%) had DM and 441 patients (13.0%) were diagnosed with TB. Among them, 236 (53.5%) patients had pulmonary TB, 185 (42.0%) had extra-pulmonary TB, and 20 (4.5%) had both pulmonary and extra-pulmonary TB simultaneously. Seven hundred patients (20.6%) had lymphocytopenia, and 1,635 (48.2%) had anemia, according to the WHO guidelines. Less than 5% of the patients had experienced chronic renal failure with renal replacement therapy, had undergone solid organ or bone marrow transplant, had HIV infection or advanced liver cirrhosis, or had received systemic corticosteroids.

**Table 1:** Baseline characteristics of enrolled population.

Variables	Total (n = 3,391)
Age (years)	54.8 ± 18.1
Male	1,864 (55.0%)
IGRA results	
• Positive	1,265 (37.3%)
• Negative	1,860 (54.9%)
• Indeterminate	266 (7.8%)
Diabetes mellitus	512 (15.1%)
Chronic renal failure with renal replacement therapy	122 (3.6%)
Rheumatic disease	486 (14.3%)
Malignancy undergoing chemotherapy	265 (7.8%)
Solid organ transplant	29 (0.9%)
Bone marrow transplant	25 (0.7%)
Human immunodeficiency virus infection	96 (2.8%)
Immunosuppressive agent use	233 (6.9%)
Advanced liver cirrhosis	76 (2.2%)
Systemic corticosteroid use	143 (4.2%)
Diagnosis of tuberculosis	441 (13.0%)
• Pulmonary tuberculosis	236 (53.5%)
• Extrapulmonary tuberculosis	185 (42.0%)
• Pulmonary and extrapulmonary tuberculosis	20 (4.5%)
Lymphocytopenia	700 (20.6%)
Anemia	1,635 (48.2%)
Glucose (mg/dL)	123.2 ± 53.8
Albumin (g/dL)	3.8 ± 0.7
C-reactive protein (mg/dL)	3.7 ± 6.0

Data are expressed as number (percentage) or mean ± standard deviation.

IGRA: interferon-gamma release assay

### Factors associated with indeterminate QFT-GIT results compared with determinate results

Table 2 shows the results of the univariate and multivariable analyses of clinical factors associated with indeterminate results compared with determinate results of QFT-GIT. In univariate analysis with logistic regression analysis, the difference between the indeterminate and determinate results was statistically significant in patients who had DM, advanced liver cirrhosis, diagnosis of TB, lymphocytopenia, or anemia, or who received systemic corticosteroids. Significant differences in the mean total age, serum albumin, and CRP levels were also observed between patients with determinate and indeterminate QFT-GIT results. Multivariable analysis by logistic regression analysis revealed that systemic corticosteroid use, a diagnosis of TB, lymphocytopenia, lower serum albumin, and higher serum CRP levels were significantly associated with indeterminate QFT-GIT results. Collinearities were not observed among variables in the multivariable logistic regression analysis (VIFs < 2.1). In contrast, DM was no longer associated with indeterminate QFT-GIT results following multivariable analysis (adjusted odds ratio [OR], 0.98; 95% confidence interval, 0.69–1.41; P = 0.939).

**Table 2:** Comparison between the determinate and indeterminate QFT-GIT result groups using logistic regression analysis.

Variables	Determinate	Indeterminate	Univariate			Multivariable		
	n = 3,125	n = 266	OR	95% CI	P	adjusted OR	95% CI	P
Age (years)	54.3 ± 18.0	61.0 ± 17.3	1.0	1.0–1.0	<0.000	1.0	0.9–1.0	0.693
Male	1,722 (55.1%)	142 (53.4%)	0.9	0.7–1.2	0.588			
Diabetes mellitus	451 (14.4%)	61 (22.9%)	1.7	1.3–2.3	<0.000	0.9	0.6–1.4	0.939
Chronic renal failure with RRT	108 (3.5%)	14 (5.3%)	1.5	0.8–2.7	0.132	0.9	0.5–1.8	0.945
Rheumatic disease	448 (14.3%)	38 (14.3%)	0.9	0.6–1.4	0.982			
Malignancy undergoing chemotherapy	238 (7.6%)	27 (10.2%)	1.3	0.9–2.0	0.141			
Solid organ transplantation	27 (0.9%)	2 (0.8%)	0.8	0.2–3.6	0.849			
Bone marrow transplantation	22 (0.7%)	3 (1.1%)	1.6	0.4–5.4	0.442			
HIV infection	91 (2.9%)	5 (1.9%)	0.6	0.2–1.5	0.639			
Immunosuppressive agent use	216 (6.9%)	17 (6.4%)	0.9	0.5–1.5	0.919			
Advanced liver cirrhosis	65 (2.1%)	11 (4.1%)	2.0	1.0–3.8	0.033	0.9	0.4–2.0	0.988
Systemic corticosteroid use	125 (4.0%)	18 (6.8%)	1.7	1.0–2.9	0.033	1.8	1.0–3.2	0.045
Diagnosis of tuberculosis	424 (13.6%)	17 (6.4%)	0.4	0.2–0.7	0.001	0.2	0.1–0.4	<0.000
Lymphocytopenia	564 (18.3%)	136 (51.1%)	4.7	3.6–6.1	<0.000	2.4	1.8–3.3	<0.000
Anemia	1,426 (46.2%)	209 (78.6%)	4.2	3.1–5.7	<0.000	1.1	0.7–1.6	0.448
Albumin (g/dL)	3.8 ± 0.7	3.0 ± 0.7	0.2	0.2–0.3	<0.000	0.3	0.2–0.4	<0.000
C-reactive protein (mg/dL)	3.1 ± 5.1	10.9 ± 9.9	1.1	1.1–1.2	<0.000	1.0	1.0–1.1	<0.000

Data are expressed as number (percentage) or mean ± standard deviation.

QFT-GIT, QuantiFERON-TB Gold In-Tube test; OR, odds ratio; CI, confidence interval; RRT, renal replacement therapy; HIV, human immunodeficiency virus.

### Relationship between indeterminate QFT-GIT results and DM: Propensity score matching analysis

We performed propensity score matching analysis to evaluate influence of DM on indeterminate QFT-GIT result compared to determinate QFT-GIT result by calculating propensity scores using logistic regression. After matching, there were 962 matched DM-non-DM patients. There was no significant difference between DM and non-DM groups after matching with absolute standardized differences of the mean were within 0.1 for all variables in each group (Table 3). Fifty-eight patients (12.1%) of DM group had indeterminate QFT-GIT results, and 60 (12.5%) had indeterminate QFT-GIT results. Logistic regression analysis revealed that DM was not associated with an indeterminate QFT-GIT result (Table 4).

**Table 3:** Patient characteristics before and after propensity score matching.

Variables	Overall cases			Propensity score-matched pairs		
	Non-diabetes	Diabetes	P	Non-Diabetes	Diabetes	P
	n = 2,879	n = 512		n = 481	n = 481	
Age (years)	53.0 ± 18.3	64.7 ± 12.9	<0.000	66.0 ± 14.2	65.0 ± 12.8	0.085
Male	1,553 (53.9%)	311 (60.7%)	0.004	282 (58.6%)	294 (61.1%)	0.454
Chronic renal failure with RRT	67 (2.3%)	55 (10.7%)	<0.000	34 (7.1%)	44 (9.1%)	0.100
Rheumatic disease	435 (15.1%)	51 (10.0%)	0.002	55 (11.4%)	50 (10.4%)	0.682
Malignancy receiving chemotherapy	221 (7.7%)	44 (8.6%)	0.475	42 (8.7%)	43 (8.9%)	1.000
Solid organ transplant	19 (0.7%)	10 (2.0%)	0.008	7 (1.5%)	7 (1.5%)	1.000
Bone marrow transplant	20 (0.7%)	5 (1.0%)	0.571	2 (0.4%)	5 (1.0%)	0.453
Advanced liver cirrhosis	53 (1.8%)	23 (4.5%)	0.001	22 (4.6%)	22 (4.6%)	1.000
HIV infection	88 (3.1%)	8 (1.6%)	0.060	5 (1.0%)	6 (1.2%)	1.000
Immunosuppressive agent use	212 (7.4%)	21 (4.1%)	0.006	19 (4.0%)	21 (4.4%)	0.871
Systemic corticosteroids use	127 (4.4%)	16 (3.1%)	0.232	10 (2.1%)	15 (3.1%)	0.424
Diagnosis of tuberculosis	363 (12.6%)	78 (15.2%)	0.116	520(10.8%)	70 (14.6%)	0.099
Lymphocytopenia	563 (19.9%)	137 (26.9%)	<0.000	127 (26.4%)	129 (26.8%)	0.942
Anemia	1,273 (44.7%)	362 (71.1%)	<0.000	331 (68.8%)	343 (71.3%)	0.290
Albumin (g/dL)	3.8 ± 0.7	3.4 ± 0.7	<0.000	3.4 ± 0.7	3.4 ± 0.7	0.527
C-reactive protein (mg/dL)	3.6 ± 5.8	5.5 ± 7.1	<0.000	5.4 ± 6.7	5.5 ± 7.2	0.809

Data are expressed as number (percentage) or mean ± standard deviation. The Pearson’s χ^2^ or Fisher’s exact test was used for categorical variables and the Student’s t-test was used for continuous variables to compare between determinate and indeterminate groups in the overall cases.

RRT, renal replacement therapy; HIV, human immunodeficiency virus

**Table 4:** Relationship between indeterminate QFT-GIT results and diabetes mellitus using multivariable logistic regression and propensity score matched analysis.

Model	No.	OR	95% CI	P value
Unadjusted	3,391	1.76	1.30–2.38	< 0.000
Multivariable ^a^	3,391	0.99	0.69–1.42	0.984
Matched on propensity score	962	0.96	0.65–1.41	0.962

QFT-GIT, QuantiFERON-TB Gold In-Tube test; OR, odds ratio; CI, confidence interval.

^a^Selected variables included age, sex, chronic renal failure with renal replacement therapy, advanced liver cirrhosis, systemic steroids, diagnosis of tuberculosis, lymphocytopenia, anemia, and albumin and C-reactive protein levels.

## Discussion

This cohort study included 3,391 subjects, which, to our knowledge, makes it the largest to date to demonstrate a relationship between DM and indeterminate IGRA results. We found that DM was not associated with indeterminate QFT-GIT results.

Indeterminate IGRA results can be caused by either a high background response or a poor response to the positive control mitogen (low mitogen). However, most reports show that indeterminate results of QFT-GIT result from low mitogen [16–19]. In a previous study conducted in our institution, it was reported that 93% of the indeterminate results were because of low mitogen, which is similar to the results of the present study [20]. Therefore, indeterminate results of QFT-GIT could be associated with immunosuppression [20–25]. In DM, dysfunctional innate and adaptive immune responses to *Mtb* have been reported, and this could increase the risk of active TB [7, 8]. Consequently, the IFN-γ response to the *Mtb* antigen may be affected in patients with DM. However, inconsistent results have been reported about this issue in TB or latent TB infection [9–12]. Variable sensitivity has been reported in QFT-GIT results among DM patients. Some studies have reported the high sensitivity of QFT-GIT in TB patients with DM [26–28]; however, a recent study reported the low sensitivity of this test in TB patients with DM [29]. In TB patients with DM, it also unclear if DM affects the incidence of indeterminate QFT-GIT results. Some studies have shown that DM was not a risk factor for indeterminate QFT-GIT results [20, 22, 23]. However, these studies had fewer than 700 enrolled patients, among whom there were fewer than 70 DM patients. Moreover, some studies focused on specific populations, such as immunocompromised patients and those with rheumatic diseases [20, 22]. A recent, large multicenter study that included our institution enrolled 1,264 patients with active TB and 203 TB patients with DM; it showed that DM was a risk factor for indeterminate QFT-GIT results (OR, 2.58; 95% CI, 1.20–5.54) [25]. However, in that study, the number of patients with indeterminate QFT-GIT results was only 40; this might cause over-fitting, and there may have been inter-dependent covariates in the multivariate analysis. Our study was strengthened by the large number of enrolled patients and the use of propensity score matching analysis. We enrolled 3,391 subjects to determine whether DM could increase indeterminate results in QFT-GIT. We also used propensity score matching to adjust possible variables affecting indeterminate IGRA results. After propensity score matching, DM was not associated with indeterminate IGRA results compared with determinate group.

In the present study, systemic corticosteroid use was associated with indeterminate IGRA results after multivariate analysis. However, other immunosuppressive conditions, including HIV infection, solid organ transplant, bone marrow transplant, advanced liver cirrhosis, and chronic renal failure with renal replacement therapy, were not associated with indeterminate IGRA results. This result might arise from the small numbers of enrolled patients with each immunosuppressive condition; less than 5% of the total study population had each condition.

Our study has some limitations. As this study was conducted retrospectively, blood glucose levels at the time of QFT-GIT testing, HbA1c, and antidiabetic therapies in DM patients were not collected. Therefore, the relationship between blood glucose control and indeterminate results of QFT-GIT in DM patients could not be proved in this study. Quantitative QFT-GIT results and CD4+ cell counts in patients with HIV infection were not assessed. Second, we could not evaluate other possible risk factors associated with indeterminate QFT-GIT results, such as specimen collection, long delays in specimen processing, incubator malfunctions, or technical errors [1].

In conclusions, this observational study used propensity score-matched cohorts to demonstrate that DM does not affect the incidence of indeterminate results in IGRA testing.
